# New onset heart failure with reduced ejection fraction management: single center, real-life Tunisian experience

**DOI:** 10.1186/s43044-023-00417-7

**Published:** 2023-11-07

**Authors:** Meriem Drissa, Marouan Krid, Fares Azaiez, Essia Mousli, Soumaya Yahyaoui, Cyrine Aouji, Habiba Drissa

**Affiliations:** 1 Cardiology Department, La Rabta University Hospital Tunis, Tunis, Tunisia; 2 Cardiology Department, Mongi Slim University Hospital Tunis, Tunis, Tunisia

**Keywords:** Chronic heart failure, Prognosis, Treatment

## Abstract

**Background:**

Heart failure (HF) is a serious and frequent pathology. It represents a major public health problem. We have few data about this pathology in our country. The aim of our study is to determine the epidemiological, clinical, therapeutic, and prognostic characteristics of new-onset HF with reduced left ventricular ejection fraction (HFrEF) and to study the degree of conformity of the management of HF with international recommendations.

**Results:**

Our study population includes 210 patients hospitalized for HFrEF newly diagnosed. The average age of our patients was 64 ± 12 years. A male predominance was noted with a sex ratio of 2.8. The main etiology of HF was ischemic heart disease noted in 97 patients (46.2%). The average LVEF is 33 ± 6%. The triple combination (angiotensin-converting enzyme inhibitors + beta blockers + Mineralocorticoid Receptor Antagonists) was prescribed in 75 patients (35.7%). The quadruple combination (angiotensin-converting enzyme inhibitors + beta blockers + Mineralocorticoid Receptor Antagonists + Sodium-Glucose Co-Transporter 2 inhibitors) was prescribed in 17 patients (8.1%). Myocardial revascularization was indicated in 97 patients (46.6%) and valve surgery was indicated in 49 patients (23.3%). Hospital mortality was 3.8% and at 1 year 18.1%. Among the 192 patients followed during the first year after discharge from hospital, 81 patients had to be re-hospitalized, i.e., a 1-year rehospitalization rate of 42.2%.

**Conclusions:**

Our study highlighted the epidemiological and clinical features of HF in a Tunisian care center, revealing our patient management deficiency. This pushes us to have a new Tunisian register to enable a better statistical analysis and lead to more relevant conclusions.

## Background

Heart failure (HF) represents a major public health problem because of its frequency, its consequences in terms of morbidity and mortality, and its considerable economic impact on the healthcare system [1]. Its prognosis in recent years has been characterized by a constant increase in its global prevalence which is between 1 and 2% in industrialized countries [2]. It remains a topical subject because of the enormous therapeutic progress made in these recent years. The management of Heart Failure with Reduced Ejection Fraction (HFrEF) is currently well codified [3] and is based on medical treatment that includes the use of 4 drugs: beta-blockers, angiotensin-converting enzyme inhibitors, sodium-glucose cotransporter 2 inhibitors, and MRAs (Mineralocorticoid Receptor Antagonists). Despite the considerable progress, the morbidity and mortality of HF remain high. The earlier the diagnosis of heart failure is made and treatment is initiated, the better the prognosis. That's why recommendations emphasize the early initiation of heart failure treatment [3]. The literature is abundant concerning HFrEF but we have few data about this pathology in our country outside our national register NATURE-HF [4] which was conducted before the emergence of the new heart failure drugs.

## Objectives

We aim to determine the epidemiological, clinical, therapeutic, and prognostic characteristics of newly diagnosed HFrEF in a Tunisian center and to study the degree of conformity of the management of HF to recent ESC guidelines [3].

## Methods

### Study patients

Our study included patients hospitalized for newly diagnosed HFrEF from April 2020 to March 2022. It is a prospective longitudinal monocentric study. HFrEF was defined as a left ventricle ejection fraction of <  = 40% on echocardiography. All patients aged over 18 years with newly diagnosed HFrEF, regardless of etiology, were included. Patients with acute myocarditis or pure right heart failure and those with heart failure with mildly reduced ejection fraction (HFmrEF) and heart failure with preserved ejection fraction (HFpEF) were excluded. The diagnosis of myocarditis was ruled out through cardiac Magnetic Resonance Imaging (MRI) when necessary. Patients with a previous diagnosis of HF were also excluded.

We recorded baseline characteristics, including age, gender, comorbidities, clinical signs, electrocardiogram results, echocardiography assessments, and management approaches. We also noted in-hospital outcomes and precipitating factors of HF episode exacerbations. After hospital discharge, patients were followed up at 3 months, 6 months, and 12 months in our outpatient clinic. An ECG was recorded during each visit, and an echocardiography was performed at the 12-month follow-up. We documented the occurrence of major events at 1 year, including cardiac death (progressive heart failure and/or sudden death) and/or re-hospitalization for heart failure. Since the study was conducted during the COVID-19 pandemic, a rapid antigen test was performed before hospitalization, and temperature measurements were taken before each consultation after hospital discharge.

Renal deficiency was defined as a creatinine clearance of <  = 60 ml/min, and anemia was defined as Hemoglobin < 13 in men and < 12 in women.

All patients provided informed consent before participating in the study.

The primary study endpoint was the occurrence of cardiac death and/or re-hospitalization for heart failure within 1 year.

### Statistical analysis

We calculated simple frequencies and relative frequencies (percentages) for the qualitative variables. We calculated means and standard deviations and determined extreme values for quantitative variables. The comparisons of the means were carried out using the ANOVA test and the Student's test for paired samples. Percentage comparisons were made by Pearson's chi-square test.

Data were analyzed using Statistical Package for Social Science (SPSS 20) software. It is a sophisticated data analysis and processing software designed by IBM. It belongs to the category of statistical analysis software.

## Results

**Fig. 1:**
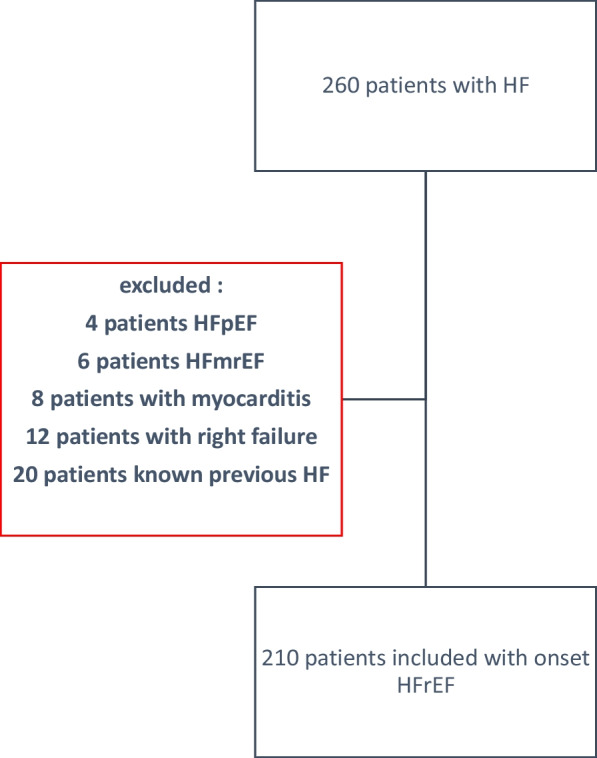
Flowchart of the study

Our study population includes 210 patients hospitalized for HFrEF newly diagnosed. The inclusion of patients was spread over 2 years from April 2020 to March 2022. The flow chart is represented in Fig. 1.

Baseline Characteristics are summarized in Table 1. The average age of our patients was 64 ± 12 years [35–85]. A male predominance was noted with a sex ratio of 2.8.

**Table 1:** Baseline characteristics

	N = 210
Male, n (%)	155 (73.8)
Smoking, n (%)	105 (50)
Hypertension, n (%)	91 (43.3)
Diabetes mellitus, n (%)	89 (42)
Dyslipidemia, n (%)	63 (30)
Chest pain, n (%)	88 (42)
Heart rate > 70 bpm, n (%)	201 (95)
*Arterial pressure*	
Systolic, mmHg	115 ± 22
diastolic, mmHg	73 ± 13
Congestive heart failure, n (%)	136 (64.7)
AF, n (%)	84 (40)
NSVT, n (%)	6 (2.8)
Ventricular premature beats, n (%)	64 (30)
LBBB, n (%)	60 (28.6)
LBBB < 120ms, %	45
LBBB [120–130], %	38.4
LBBB [130-150ms], %	11.6
LBBB > 150ms, %	5
Q wave, n (%)	21 (19)
Hemoglobin level, g/dl	12 ± 1
Creatinine level, ùmol/l	97.1 ± 32.4
LVEF < 35%, n (%)	130 (61.9)
RV dysfunction, n (%)	21 (10)
*Causes of HFrEF*	
Ischemic cause, n (%)	97 (46.2)
Valvular cause, n (%)	50 (23.8)
Idiopathic, n (%)	34 (16.2)
Hypertensive, n (%)	29 (13.8)
ACEi, n (%)	182 (86,6)
BB, n (%)	186 (88,6)
MRA, n (%)	75 (35,7)
Furosemide, n (%)	210 (100)
Ivabradine, n (%)	10 (21)
SGLT2i, n (%)	19 (9)
Sacubitril/Valsartan, n (%)	31 (15)
ACEI + BB + MRA + SLGT2i, n (%)	17 (8.1)
ACEI + BB + MRA, n (%)	75(35.7)
ACEI + BB, n (%)	164 (78)
ACEI or BB, n (%)	30 (14.3)
CRT-P, n (%)	10 (4.8)
CRT-D, n (%)	6 (2.8)
ICD, n (%)	8 (3.8)

AF, atrial fibrillation; NSVT, non-sustained ventricular tachycardia; LBBB, left bundle branch block; LVEF, left ventricle ejection fraction; RV, right ventricle; HFrEF, heart failure with reduced ejection fraction; ACEi, angiotensin-converting enzyme inhibitors; BB, Beta-blockers; MRA, mineralocorticoid receptor antagonists; SGLT2, Sodium-glucose cotransporter 2 inhibitors; CRT-P, cardiac resynchronization therapy with pacemaker; CRT-D, cardiac resynchronization therapy with defibrillator; ICD, implantable cardioverter defibrillator

50% of patients were smokers, 43.3% hypertensive, 42% diabetic and 30% dyslipidemic.

Anemia and renal deficiency were the most observed comorbidities respectively in 60 patients (28.5%) and 48 patients (22.8%). In our series, ferritin level measurement was not systematically performed in all patients.

Left bundle branch block (LBBB) was noted in 60 patients (28.5%) with an average QRS duration of 126.8 ± 16.6 ms [120–160].

On echocardiography, the left ventricle ejection fraction (LVEF) was on average 33 ± 6% [15–40].

The main etiology of HF was ischemic heart disease noted in 97 patients (46.2%) followed by valvular heart disease in 50 patients (23.8%), primary dilated cardiomyopathy in 34 patients (16.2%), and hypertensive heart disease in 29 patients (13.8%).

The most commonly used medications were loop diuretics (100% of patients), angiotensin-converting enzyme inhibitors (ACEi) (86.6%), Beta-blockers (BB) (88.6%), and mineralocorticoid receptor antagonists (MRA) (35.7%). 19 patients were on Sodium-glucose cotransporter 2 inhibitors (SGLT2i), 31 patients were on Sacubitril/Valsartan and 10 patients were on Ivabradine.

Bisoprolol was prescribed in all cases. The initial dose was on average 1.78 ± 0.6 mg [1.25–2.5] and the maximum tolerated dose was on average 4.68 ± 1.9 mg [1.25–10]. The maximum recommended dose was only reached in 30 patients (16%).

Captopril was prescribed in all cases. The initial dose was on average 35 ± 17.8 mg [12.5–100 mg]. The maximum tolerated dose was 55.6 ± 31 mg [12.5–150 mg]. The maximum recommended dose was reached in only 21 patients (10%).

The maximum tolerated dose of MRA was reached in 15 patients (20%).

The triple combination ACEI + BB + MRA was prescribed in 75 patients (35.7%). The quadruple combination (ACEI + BB + MRA + SLGT2i) was prescribed in 17 patients (8.1%). Myocardial revascularization was indicated in 97 patients (46.6%) and valve surgery was indicated in 49 patients (23.3%).

Cardiac resynchronization therapy (CRT) was performed in 10 patients (4.7%). The Implantable Cardioverter Defibrillator (ICD) was performed on 5 patients who did not meet the resynchronization criteria. The implantation of a cardiac resynchronization therapy with defibrillator (CRT-D) was performed in 6 patients (2.8%). We noted no case of cardiac rehabilitation or heart transplantation.

### Study outcomes

#### Intra-hospital evolution

The average length of hospital stay was 32 ± 2 days [16–45 days]. During the hospital stay, hospital mortality ranged about 3.8%. The time to death was on average 20 ± 4 days [16–26 days]. A clinical improvement was obtained in 202 patients (96.2).

#### Evolution at 1 year

The follow-up concerned 192 patients because 10 patients did not undergo follow-up during the 1st year.

30 patients died during the first year after discharge. The total number of cardiovascular deaths compared to inclusion was 38 patients (18.1%). The mean time to death was 234 ± 29 days [55–320].

**Fig. 2:**
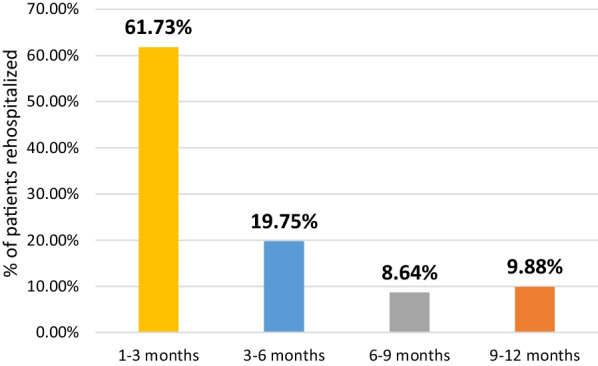
Rehospitalization rate during the 1-year follow-up period

**Fig. 3:**
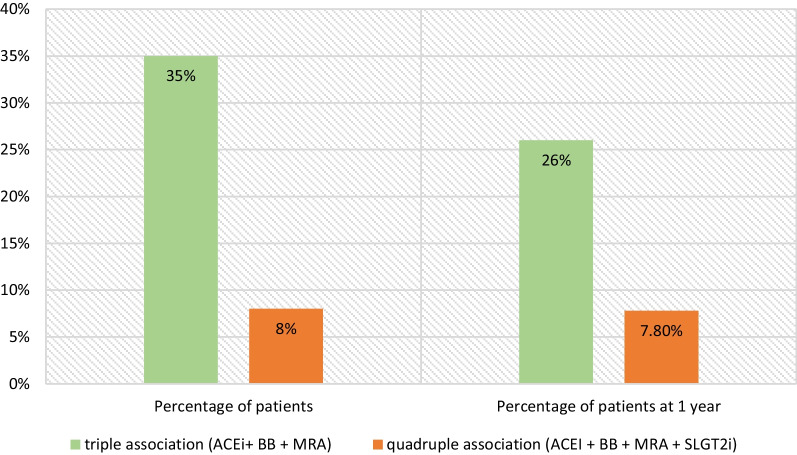
Number of patients at baselines and at 1 year of follow-up under medical treatment

Among the 192 patients followed during the first year after discharge from hospital, 81 patients had to be re-hospitalized, i.e., a 1-year rehospitalization rate of 42.2%. The mean time to re-hospitalization was 115 ± 45 days [24–360]. The rehospitalization rate during the 1-year follow-up period is shown in Fig. 2. Figure 3 represents the evolution of the number of patients under medical treatment after 1 year of follow-up.

The most common precipitating factor for decompensation was bronchopulmonary infection observed in 105 patients (41%), followed by anemia in 52 patients (22%), an ischemic episode in 42 patients (20%), flutter or atrial fibrillation in 25 patients (12%), urinary tract infection in 15 patients (7%), and pulmonary embolism (PE) in 5 patients (2%).

## Discussion

Our study highlighted the epidemiological and clinical features of HF in a Tunisian care center. This pathology mainly affects elderly and male subjects. Patients are seen at an advanced stage of HF. We noted a predominance of ischemic etiology, and a satisfactory rate of the triple combination (BB + MRA + ACEi). However, the optimal doses were not reached, and the prescription of new molecules recently prescribed (SGLT2i and Sacubitril/Valsartan) was low and few patients were on the 4 molecules as recommended in ESC guidelines. It also revealed our deficiency in patient care, in particular the readaptation and the realization of cardiac transplantation. We demonstrate also a poor prognosis with huge mortality and iterative rehospitalizations.

The mean age of heart failure was 70 years in Framingham study [5]. Other studies reported an average age of patients at the time of HF diagnosis of 65–75 years [6–9]. In our series, the average age of our patients was comparable to that reported in these different studies: 64 years. It was similar to the average age found in our NATURE-HF register with an average age of 63.6 years [4].

The predominance of males was reported in several studies [10, 11]. In our study, 73.8% were male which is concordant with the results of NATURE-HF (70.9%) [4] and the European Society of Cardiology Heart Failure Long-Term Registry (ESC-HF-LT)(71.2% for chronic heart failure and 62.6% for acute heart failure) [12].

The major cardiovascular diseases associated with heart failure were smoking, hypertension, and diabetes. In fact, two-thirds of patients with HF have a history of hypertension [12]. Using data from the Framingham cohort, Levy [13] showed that the risk of heart failure was mainly linked to hypertension and myocardial infarction in men, and to myocardial infarction and diabetes in women. In our study, we found a rate of 48% of hypertensive patients joining our national register which reveals a rate of 42.1% [4]. Diabetic patients were at risk of developing heart failure regardless of coronary lesions [14]. The Framingham study showed that diabetic patients were twice at risk of developing HF in men and five times at risk in women [5]. In our NATURE-HF registry, the rate of diabetic patients was 37, 8% [4] and in our series, we found a high rate of 48%. 50% of patients were smokers in our study, and a lower rate was found in our NATURE-HF register [4] (27.3%) and in ESC-HF-LT (11.2% for chronic heart failure and 16% for acute heart failure) [12].

In the literature, the average HR reported was 82 ± 21 according to Gotsman [15] and 93.5 ± 25 according to Macin [16], It was 90.82 ± 25.27 for acute heart failure and 72.7 ± 15.29 for chronic heart failure in ESC-HF-LT [12] and 96 ± 20 bpm in our study series joining our NATURE-HF registry where the mean HR was around 80 ± 17 bpm [4]. AF is the most common arrhythmia seen in heart failure. A frequency ranging from 23 to 40% has been reported in the literature [17, 18]. It was observed in 40% of patients in our series, 30.6% according to our NATURE-HF register [4], 44% in acute heart failure, and 37.7% in chronic heart failure in ESC-HF-LT [12].

LBBB was a frequently noted sign during heart failure [19]. It was observed in 40.6% of patients in Macin's study [16]. We reported a rate of 28.5% in our study. It was higher than the rate in the NATURE-HF register, which was around 11.1% [4].

In our study, 28.5% of patients were anemic, approaching the rate found in our NATURE-HF register, which was 23.6% [4] while 47% in REPORT-HF [20].

In Western countries, coronary disease was the most common cause of heart failure in 60–70% of cases [21]. It is followed by hypertensive disease (20–30%), cardiomyopathy (5–10%), and valvular disease (3–10%).

In our NATURE-HF registry, ischemic etiology was observed in 52.4% followed by primary DCM with a rate of 15.5%, valvular heart disease with 9.9%, and HCM 1.3% [4].

In ESC-HF-LT [12], ischemic heart disease was seen in 53.8% of acute heart failure and in 43% of chronic heart failure, and in 48% in REPORT-HF [20].

The prevalence of renal deficiency in patients with heart failure was around 26% in ESC-HF-LT [12]. This is also found in our series where 22.8% of patients had renal failure concordant with the rate found in our NATURE-HF register: 25% [4].

Over the last 20 years, HF management has been codified thanks to recent updates of European recommendations of 2021 and the American guidelines. In our study, the most commonly used drugs were diuretics (mainly furosemide), angiotensin-converting enzyme inhibitors (ACE-I), beta-blockers (BB), mineralocorticoid receptor antagonists (MRA), Sodium-glucose cotransporter 2 inhibitors (SGLT2i) and Sacubitril/Valsartan.

In Mahler's study including patients with HFrEF from six European countries, 87% of patients received an ACE Inhibitor or an Angiotensin Receptor Blockers (ARB) [22]. Comparable rates have been observed in the multicentric study ESC-HF Pilot [23] including 5118 patients with heart failure where ACE Inhibitors or ARBs were prescribed in 88.5%, BB in 86.7%, Spironolactone in 43.7% and loop diuretics in 82.8%. Higher rates than those reported in this study were observed in our series, in fact, ACE Inhibitor or ARBs were prescribed in 86.6%, BB in 88.6%, and Spironolactone in 35, 7%. In our National nature-HF register, there were rates similar to our study with ACE inhibitors prescribed at 80.5%, BB at 80%, and Spironolactone at 50.5% [4]. According to the REPORT-HF [20], 70% of patients were on ACE inhibitors, 86% on loop diuretics, 76% on BB, and 59% on MRA.

In our study, the triple combination ACEI + BB + MRA was prescribed in 75 patients (35.7%). The quadruple combination (ACEI + BB + MRA + SLGT2i) was prescribed in 17 patients (8.1%).

The prescription of drugs for HF is not optimal as the prescription of the triple combination was only 35% in the ESC-HF Pilot [23] and the number of patients treated with appropriate doses was low. Different factors have been identified to explain this including age, sex, and comorbidities, especially renal deficiency. In our study, the recommended optimal doses for each class were reached only in 21 patients (10%) for ACE-I, in 30 patients (16%) for BB, and in 15 patients (20%) for MRA. ACEi/ARB/ARNi was prescribed in optimal dose in 22% of low-income countries vs. 28% in high-income countries β-blockers in 7% of low-income countries vs. 32% in high-income countries and MRA in 14% of low-income countries vs. 9% in high-income countries in the REPORT-HF [24]. The non-optimal treatment was explained in our study by arterial hypotension, worsening renal function, and exacerbation of heart failure symptoms in some patients.

In our study, only Captopril and Bisoprolol were used as drugs. This can be explained by the fact that these two drugs are provided by the hospital and are part of the hospital formulary.

Despite their effect proven in several studies on mortality and rehospitalization in heart failure [25, 26], we noted a reduced prescription of Sodium-glucose cotransporter 2 inhibitors (SGLT2i) or Sacubitril/Valsartan. Besides, in the Nature-HF register, 0.2% of patients were on Sacubitril/Valsartan, and no patients were on SGT2i [4].

This can be explained by the fact that the registry was conducted before the introduction of SGLT2i in our country and Sacubitril/Valsartan was newly introduced.

The number of patients under quadruple combination and triple combination decreased after 1-year follow-up from 8 to 7.8% and from 35 to 26% respectively. 37% of patients were on the triple combination at discharge and 34% at 6 months of follow-up in the REPORT-HF [24].

Non-pharmacological treatment that can improve the prognosis (resynchronization and defibrillator) remains weak. Indeed, in our study cardiac resynchronization was performed only on 30 patients (9%) and implantable cardioverter defibrillator on 15 patients; The explanation for the non-implantation of these devices were: patient refusal, problems of logistics and cost, and low percentage of patients eligible for resynchronization. Cardiac transplantation is not often performed due to its high economic cost and its logistical problems. No case of transplantation was reported in our study.

Strong evidence from clinical trials and meta-analyses indicates that physical training improves exercise tolerance and quality of life in patients with heart failure. Moreover, several meta-analyses suggest that it reduces hospitalizations for all causes, including heart failure, although uncertainty remains regarding its effects on mortality [27].

In the Framingham study [5], which included 652 subjects, the median survival duration was 1.66 years for men and 3.17 years for women. Survival rates at 1, 2, 5, and 10 years were 57%, 46%, 25%, and 11%, respectively, for men, and 64%, 56%, 38%, and 21%, respectively, for women. The age-adjusted mortality rate, or more precisely, fatality rate, was lower in women than in men. Mortality rates increased with age in both sexes, by 27% per decade in men and 61% per decade in women.

In our study, hospital mortality was around 3.8%, comparable to that reported in other registers where it varied from 4 to 7% [28]. The Alarm-HF study [29] reported higher hospital mortality (11%).

Mortality at 1 year was around 19.8% in our study, 13% in our National Nature-HF register [4], and 23.6% among acute heart failure patients in ESC-HF-LT [12]. It was 20% in REPORT-HF [20].

The rate of readmission at 1 year in our study was around 42.2%, this rate is almost comparable to that noted in the Astronaut register: 27.6% in North America and 22.5% in Western countries [30].

Many studies have reported the ischemic origin of heart failure as an unfavorable prognostic factor [31]. We should note that precipitating factors can influence rehospitalization and mortality rates.

Our study has demonstrated that the distribution of readmissions over 1 year is unequal, with the highest rate occurring in the first 3 months at 61.73%, and this rate decreases over time. Similar findings have been observed in other studies: Wideqvist et al. found that 60.1% of readmission rates occurred in the first quarter, 17.2% in the second quarter, 15.4% in the third quarter, and 7.3% in the fourth quarter [32].

This rate of mortality can be explained by the lack of use of new drugs which has demonstrated their efficacy in reducing mortality and rehospitalizations, in addition to the non-optimization of doses of medical treatment, the weak implantation of devices, and the absence of cardiac rehabilitation.

## Limitations

The most important limitation was the monocentric study character with a reduced number.

Despite these limitations, our study constitutes the first Tunisian study that is interested in studying the current profile of our patients as well as their prognosis for the last 2 years 2020 2022 since the use of new therapies because the nature-HF register is old carried out in 2019 before the publication of the recommendations of ESC.

This pushes us to have a second Tunisian register to enable better statistical analysis and lead to more relevant conclusions to determine the exact therapeutic profile of our patients and their prognosis under new therapies.

## Conclusions

This study outlined the epidemiological, clinical features and outcomes of HF in a Tunisian center, revealing our patient management deficiency. The results encourage us to create the heart failure therapeutic unit to optimize the treatment according to what is recommended to take further preventive measures to improve the prognosis.

The best treatment for HFrEF is its prevention based on early diagnosis, and optimal and effective management of the causal pathology before the deterioration of left ventricular function.

## Data Availability

The authors declare the availability of data used in the study.

## Abbreviations

ACEi: Angiotensin-converting enzyme inhibitors
AF: Atrial fibrillation
ARBs: Angiotensin receptor blockers
BB: Beta-blockers
CRT-D: Cardiac resynchronization therapy with defibrillator
CRT-P: Cardiac resynchronization therapy with pacemaker
HF: Heart failure
HFmrEF: Heart failure with mildly reduced ejection fraction
HFpEF: Heart failure with preserved ejection fraction
HFrEF: Heart failure with reduced ejection fraction
ICD: Implantable cardioverter defibrillator
LBBB: Left bundle branch block
LVEF: Left ventricle ejection fraction
MRA: Mineralocorticoid receptor antagonists
NSVT: Non-sustained ventricular tachycardia
p: Patients
RV: Right ventricle
SGLT2: Sodium-glucose cotransporter 2 inhibitors
